# Antimicrobial Resistance in *Escherichia coli* and Enterococcal Isolates From Irrigation Return Flows in a High-Desert Watershed

**DOI:** 10.3389/fmicb.2021.660697

**Published:** 2021-05-12

**Authors:** Robert S. Dungan, David L. Bjorneberg

**Affiliations:** Northwest Irrigation and Soils Research Laboratory, Agricultural Research Service, United States Department of Agriculture, Kimberly, ID, United States

**Keywords:** antimicrobial susceptibility, *Escherichia coli*, enterococci, irrigation return flow, fecal indicator bacteria, watershed

## Abstract

Irrigation return flows (IRFs) collect surface runoff and subsurface drainage, causing them to have elevated contaminant and bacterial levels, and making them a potential source of pollutants. The purpose of this study was to determine antimicrobial susceptibility among *Escherichia coli* and enterococcal isolates that were collected from IRFs in a south-central Idaho watershed. Environmental isolates can be a potentially important source of antimicrobial resistance (AMR) and IRFs may be one way resistance genes are transported out of agroecosystems. Water samples were collected from nine IRFs and one background site (canal water from Snake River) on a biweekly basis during 2018. *Escherichia coli* and enterococci were enumerated *via* a most probable number (MPN) technique, then subsamples were plated on selective media to obtain isolates. Isolates of *E. coli* (187) or enterococci (185) were tested for antimicrobial susceptibility using Sensititre broth microdilution plates. For *E. coli*, 13% (25/187) of isolates were resistant to tetracycline, with fewer numbers being resistant to 13 other antimicrobials, with none resistant to gentamicin. While 75% (141/187) of the *E. coli* isolates were pan-susceptible, 12 multidrug resistance (MDR) patterns with 17 isolates exhibiting resistance to up to seven drug classes (10 antimicrobials). For the enterococcal species, only 9% (16/185) of isolates were pan-susceptible and the single highest resistance was to lincomycin (138/185; 75%) followed by nitrofurantoin (56/185; 30%) and quinupristin/dalfopristin (34/185; 18%). In addition, 13 enterococcal isolates belonging to *Enterococcus faecalis*, *Enterococcus faecium*, *Enterococcus casseliflavus*, and *Enterococcus thailandicus*, were determined to be MDR to up to six different antimicrobial drug classes. None of the enterococcal isolates were resistant to gentamycin, linezolid, tigecycline, and vancomycin.

## Introduction

Antimicrobials are used as the first line of defense in treating bacterial infections, greatly reducing morbidity and mortality associated with diseases in humans and animals. However, antimicrobial resistance (AMR) in bacteria is on the rise and a growing threat to the future welfare of countries around the world if not mitigated (O’Neill, 2016). In agroecosystems, the prevalence of AMR is often attributed to questionable livestock production practices that began in the 1950s, such as use of subtherapeutic amounts of antimicrobials in feed or water for the purpose of enhancing the production performance of livestock (Franklin et al., 2016). For example, the majority of *Escherichia coli* in United Kingdom swine herds had become tetracycline-resistant after 18 years of feeding low doses of this antimicrobial (Smith, 1970). Chickens fed chlortetracycline, either intermittently at a high concentration or at a low concentration throughout life, were found to carry tetracycline-resistant *Enterococcus faecalis* (Elliott and Barnes, 1959). While livestock production practices like this are certainly a driving force in the evolution of antimicrobial-resistant bacteria (ARB), AMR is an ancient bacterial trait that did not originate as a product of agricultural antimicrobial use (Allen and Stanton, 2014). Nonetheless, a temporal analysis of archived agricultural soils from 1940 to 2008 in Netherlands suggests that the antimicrobial era is responsible for increasing environmental AMR, as the number of antimicrobial resistance genes (ARGs) from major antimicrobial classes significantly increased during that period (Knapp et al., 2010). In agricultural soils, the application of manure, wastewater, or biosolids have been demonstrated to increase ARG levels, but not under all conditions (Marti et al., 2013; Rothrock et al., 2016; Dungan et al., 2018; McKinney et al., 2018). It is also important to consider that ARGs are likely horizontally transferred from manure-borne bacteria to indigenous soil bacteria (Smalla et al., 2000), thus increasing the persistence of resistance genes in soil (Binh et al., 2008; Heuer et al., 2011).

Both *E. coli* and enterococci are used as indicator organisms of fecal pollution in surface waters because they are highly abundant, easy to culture, and widely associated with the mammalian intestinal tract. Although fecal indicator bacteria (FIB) may not directly cause human illness, high levels in recreational waters were found to be associated with an increased risk for gastrointestinal illness (United States EPA, 1986). In a rural mixed-use watershed, FIB enter surface waters from human and non-human sources through a variety of pathways, which could be on a continuous basis, such as discharges from a wastewater treatment plant or failing septic system, episodically during storm or irrigation events, or by direct defecation by animals into waterways. In an agricultural watershed in southern Alberta, significant positive correlations were observed between FIB levels in river samples and farm size, total land area used for irrigation, and total land area used for manure application (Jokinen et al., 2012). Scott et al. (2017) found that *E. coli* levels in the Illinois River watershed in northwest Arkansas increased significantly once the percentage of pasture in the drainage area exceeded 55%. In a California agricultural watershed in the San Joaquin Valley, three irrigation return flows (IRFs) were found to exceed *E. coli* and enterococci standards in approximately 50 and 100% of water samples collected, respectively (Diaz et al., 2010). Runoff from fields does contribute a significant load of fecal bacteria to waterways, increasing the threat of pathogens in water supplies used by humans for recreational, irrigation, and consumption purposes (Ferguson et al., 2003; Jokinen et al., 2012). However, FIB can survive for extended periods in stream sediments and become resuspended in the water column when the sediments are disturbed (Jamieson et al., 2003; Rehmann and Soupir, 2009; Cho et al., 2010; Brinkmeyer et al., 2015). As a result, when water samples test positive for FIB, it could either be an indication of fecal contamination or that naturalized extraintestinal populations have been released from soils or streambed sediments (Ishii et al., 2006; Field and Samadpour, 2007).

There is growing evidence that the environment is an important reservoir and source of ARGs for bacteria infecting humans and animals, and that manure-impacted water may be one way that antimicrobials, resistant bacteria, and resistance genes are transported out of agroecosystems (Pruden et al., 2012; Rothrock et al., 2016; Dungan et al., 2017b). Given that *E. coli* and enterococci are of fecal origin, can adapt to live for extended periods in the environment, and can be opportunistic or true pathogens causing a variety of illnesses in humans, their role with respect to AMR in aquatic environments is worthy of investigation. Potential human exposures can occur when fecally contaminated surface waters are used for irrigation of crops or recreational purposes. Various studies to date have investigated antimicrobial resistant *E. coli* and *Enterococcus* spp. in food, animals and humans (Bell et al., 1998; Klein, 2003; Jackson et al., 2011; Frye and Jackson, 2013; Adenipekun et al., 2015; Agga et al., 2016), with some consideration of isolates from surface waters (Chen et al., 2016; Gomi et al., 2016; Cho et al., 2020b) and other related environmental matrices (Sayah et al., 2005; Ibekwe et al., 2011; Maal-Bared et al., 2013). In the present study, our objective was to increase knowledge of the abundance and AMR of *E. coli* and enterococci in IRFs the Upper Snake River watershed in south-central Idaho. The IRFs, which return excess irrigation water back to the Snake River, as well as subsurface drainage and surface runoff, represent a potential conduit for the transfer of chemical and biological contaminants (Bjorneberg et al., 2015; Dungan et al., 2017b). Results from antimicrobial susceptibility tests were also used to determine the prevalence of multidrug resistance (MDR; resistance to three or more antimicrobial drug classes) among the FIB isolates.

## Materials and Methods

### Watershed in South-Central Idaho

The Twin Falls tract (820 km^2^) of the Upper Snake Rock (USR) watershed, is located along the south side of the Snake River in south-central Idaho, United States, and has been part of the USDA Conservation Effects Assessment Project (CEAP) since 2005 (Bjorneberg et al., 2008). In this watershed, water from the Snake River is used to irrigate crops, which would otherwise not grow in this semiarid region due to lack of precipitation during the crop growing season. Snake River water is diverted into canals from mid-April to late October, increasing IRF. Some IRF streams continue to flow after the irrigation season due to water flowing from subsurface drainage. Water samples were collected from eight IRF sampling sites: Cedar Draw (CD), Deep Creek (DC), Hansen Coulee (HC), I Coulee (IC), Mud Creek (MC), N Coulee (NC), Rock Creek Poleline (RCP), and Twin Falls Coulee (TFC). A sample was also collected from the Main Line Canal (MLC), which contains diverted Snake River water and supplies more than 75% of the water to the Twin Falls tract (Bjorneberg et al., 2020), and for the purposes of the present study was designated as a background site. The sampling sites were selected to match those from previous USR watershed studies (Dungan et al., 2017b; Dungan and Bjorneberg, 2020).

### Water Sample Collection

Water samples were collected on a biweekly basis from 9 January to 18 December in 2018 with some exceptions because not all sites have water outside the growing season. Specifically, samples could only be collected from NC, TFC, HC, and MCL when the irrigation water was available starting in mid-April, with the last samples collected from these sites on 23 October. Samples could be collected from DC, MC, IC, CD, and RCP, before and after the irrigation season, since these streams flow all year due to subsurface drainage. On collection day, surface water samples were collected in sterile 500 mL polypropylene bottles, then stored in a cooler until delivered to the laboratory. At the laboratory the water samples were immediately placed under refrigeration at 5°C and subsequently processed within 24 h.

### Enumeration of *Escherichia coli* and Enterococci

Water samples (100 mL) were either processed directly or diluted when necessary into IDEXX (Westport, ME, United States) Colilert or Enterolert substrate for enumeration of total coliforms/*E. coli* and enterococci, respectively, as recommended by the manufacturer. In brief, the mixtures were then transferred to an IDEXX Quanti-Tray/2000, sealed using the IDEXX Quanti-Tray Sealer PLUS, and then, respectively, incubated at 35 and 41°C for 24 h as recommended by the manufacturer. Positive control organisms, consisting of *Klebsiella pneumoniae* subsp. *pneumoniae* (ATCC [American Type Culture Collection] 13883) and *Escherichia coli* (ATCC 25922) for Colilert and *E. faecalis* (ATCC 29212) for Enterolert, were utilized to confirm the effectiveness of each new substrate lot. After the 24-h incubation period, the Colilert and Enterolert trays were viewed under a 365-nm UV light, with blue fluorescing wells marked positive for *E. coli* and enterococci. In Colilert trays, yellow wells under normal lighting were marked positive for total coliforms. The number of positive wells were converted to most probable number (MPN) using the IDEXX result interpretation table and the dilution factor.

### Isolation of *Escherichia coli* and Enterococci

The back of each Quanti-Tray/2000 was wiped with 70 % ethanol, then a flamed surgical scalpel was used to create a small opening in a positive well. A 10 μL loopful of solution from the well was then transferred to a 150 mm plate containing CHROMagar *E. coli* (CHROMagar, Paris) for the Colilert wells and m Enterococcus Agar (Becton, Dickinson and Co., Franklin Lakes, NJ, United States) for the Enterolert wells. Up to five plates were inoculated from separate wells on each tray. The CHROMagar plates were incubated at 35°C for 24 h, while m Enterococcus plates were incubated at the same temperature for up to 48 h. Afterward, a well-isolated colony (blue from CHROMagar E. coli, red from m Enterococcus) was removed from each plate and streaked for isolation. *Escherichia coli* were streaked onto 2× Yeast Extract Tryptone (2× YT) medium plus agar plates (Becton, Dickinson and Co.), while enterococci were streaked onto Brain Heart Infusion (BHI) agar plates (Becton, Dickinson and Co.), followed by incubation at 35°C for 24 h. Select colonies were then placed in 2-mL cryovials with a solution of either 2× YT or BHI broth and 10% glycerol and stored at **−**80°C.

### Preparation of DNA From Isolates

Prior to cryopreservation, a colony from all *E. coli* and enterococcal isolates was transferred to a well of a 96-well PCR plate containing 100 μL of either molecular biology grade water (Hyclone, Logan, UT, United States) for *E. coli* or Tris-EDTA buffer, pH 8.0 (Sigma-Aldrich, St. Louis, MO, United States) for enterococci. The plate was sealed with foil sealing film (Microseal F Foil, Bio-Rad) and then placed into a thermocycler and heated at 100°C for 10 min. The cellular debris was pelleted by centrifugation at 1,000 × *g* for 2 min, then the plates were stored at **−**20°C until PCR was performed. *Escherichia coli* (ATCC 25922) and *E. faecalis* (ATCC 29212) were used as positive control organisms.

### Genetic Analysis of *Escherichia coli*

The phylotyping method as modified by Doumith et al. (2012) was used to assign *E. coli* isolates into one of seven phylotypes (A0, A1, B1, B2.2, B2.3, D1, and D2) as described by Escobar-Paramo et al. (2004b). Briefly, 3 μL of template DNA was used in a multiplex PCR assay that utilized 12.5 μL of AmpliTaq Gold 360 Master Mix (Applied Biosystems, Carlsbad, CA, United States), forward and reverse primers (600 nM *chuA*, 400 nM *gadA*, 200 nM *yjaA*, and 200 nM TSPE4.CA), and molecular biology grade water to a final volume of 25 μL. DNA from *Escherichia coli* ATCC 25922 was used as template in positive controls and molecular biology grade water was used for no-template controls. The primer sequences (5′–3′) were: *chuA*-F, ATGATCATCGCGGCGTGCTG; *chuA*-R, AAACGCGCTCGCGCCTAAT; *gadA*-F, GATGAAATGGCGTT GGCGCAAG; *gadA*-R, GGCGGAAGTCCCAGACGATATCC; *yjaA*-F, TGTTCGCGATCTTGAAAGCAAACGT; *yjaA*-R, ACC TGTGA CAAACCGCCCTCA; TSPE4.CA-F, GCGGGTGAGA CAGAAACGCG; TSPE4.CA-R, TTGTCGTGAGTTGCGAAC CCG (Doumith et al., 2012). The respective amplicon lengths for *chuA*, *gadA*, *yjaA*, and TSPE4.A were 281, 373, 216, and 152 bp. The thermocycler (T100, Bio-Rad, Hercules, CA, United States) conditions consisted of (i) one initial denaturation cycle at 95°C for 10 min; (ii) 30 amplification cycles at 95°C for 30 s, 65°C for 30 s, and 72°C for 30 s; and (iii) a final extension at 72°C for 7 min. PCR products were electrophoresed on 2% agarose gels in 1 × Tris-Borate-EDTA (TBE). Afterward, the gels were stained for 30 min in a solution containing SYBR Green 1 (Invitrogen, Eugene, OR, United States), then visualized under UV (302 nm) in a Gel Doc XR+ System (Bio-Rad) and photographed using a SYBR photographic filter. A 50 bp DNA ladder (Invitrogen, Waltham, MA, United States) was used to confirm the size of amplification products.

To determine if any of the *E. coli* isolates were enterohemorrhagic, quantitative real-time PCR was utilized for the detection of Shiga toxin (i.e., *stx*1, *stx*2) and intimin (i.e., *eae*) genes as described by Dungan et al. (2012). The primer sequences (5′–3′) were: *stx1*-F, GACTGCAAAGACGTATGTAGATTCG; *stx*1-R, ATCTATCCCTCTGACATCAACTGC; *stx*2-F, ATTAA CCACACCCCACCG; *stx*2-R, GTCATGGAAACCGTT GTCAC; *eae*-F, GTAAGTTACACTATAAAAGCACCGTCG; *eae*-R, TCT GTGTGGATGGTAATAAATTTTTG.

### Identification of Enterococcal Isolates

Polymerase chain reaction was performed on the enterococci lysis products for subsequent sequencing and identification of the isolates. Individual PCR reactions consisted of 12.5 μL of Invitrogen Platinum Green Hot Start PCR 2× Mastermix (ThermoFisher Scientific, Waltham, MA, United States), 300 nM of forward (Ent-ES-211-233-F-bio; 5′-GHACAGAAGTRAAATAYGAAGG-3′) and reverse (Ent-EL-74-95-R; 5′-GGNCCTAABGTHACTTTNACTG-3′) primers (Zaheer et al., 2012), 2 mL of DNA template (lysis product), and molecular biology grade water to a final volume of 25 μL. The thermocycler conditions consisted of one cycle at 94°C for 2 min, 35 cycles of 94°C for 30 s, 51°C for 30 s and 72°C for 30 s and one cycle at 72°C for 5 min. *E. faecalis* (ATCC 29212) lysis product was run as a positive control and molecular biology grade water was run as a negative control. The PCR products were loaded directly onto 2% agarose gels and electrophoresed in 1× TBE. The gels were stained with SYBR and visualized as described above. A 50 bp DNA ladder was used to confirm the size of the amplification products, which were about 200 bp. The remaining PCR product was shipped to TACGen (Richmond, CA, United States) and subsequently pyrosequenced using the degenerate sequencing primer Ent-sp-seq (5′-GCAAATTTVAWHTCTTTTGCCAT-3′) (Zaheer et al., 2012). Raw sequences were handled and trimmed using Chromas 2.6.6 (Technelysium Pty Ltd., South Brisbane) and then checked for putative chimeric sequences using DECIPHER (Wright et al., 2012). Sequences were compared to those in GenBank using BLAST (Basic Local Alignment Search Tool) of NCBI (National Center for Biotechnology Information, Bethesda, MD, United States).

### Antimicrobial Susceptibility Testing

*Escherichia coli* and enterococcal isolates were tested for their susceptibility to a panel of 14 and 16 antimicrobials by broth microdilution using the National Antimicrobial Resistance Monitoring System (NARMS) Gram Negative CMV3AGNF and Gram positive CMV3AGPF plates (Sensititre, ThermoFisher Scientific), respectively. Prior to the Sensititre analyses, a small amount of frozen material was aseptically removed from selected isolate cryovials, then it was streaked on 2× YT and BHI agar plates for *E. coli* and enterococcus, respectively. The agar plates were incubated for 16–24 h at 35°C. Colonies were transferred to 5 mL of sterile demineralized water (Cat no. T3339, ThermoFisher Scientific), followed by vortexing until the cells were completely dispersed, then measured on a DEN-1B densitometer (Grant Instruments Ltd., Cambridgeshire) and adjusted accordingly by adding more colonies until it reached 0.5 McFarland. This suspension (10 μL) was then added into a vial containing 11 mL of Mueller-Hinton broth (Cat no. T3462, ThermoFisher Scientific) and mixed by vortexing. The vial contents were transferred to a sterile 10 mL reagent reservoir and 50 μL was transferred into each well of a Sensititre plate using an 8-channel pipette. As recommended by the manufacturer, *Escherichia coli* (ATCC 25922) and *E. faecalis* (ATCC 29212) were used as quality control organisms for CMV3AGNF and CMV3AGPF plates, respectively. After inoculation, the plates were sealed with plate film and incubated at 35°C for 18 h. The plates were read manually using a Sensititre Manual Viewbox (ThermoFisher Scientific).

Sensititre plate results were interpreted according to NARMS-established breakpoints available on the United States Food and Drug Administration website^1^. For *E. coli*, the panel of 14 antimicrobials and breakpoints for classification as resistant were as follows: amoxicillin/clavulanic acid, ≥ 32/16 μg mL**^–^**^1^; ampicillin, ≥ 32 μg mL**^–^**^1^; azithromycin, ≥ 16 μg mL**^–^**^1^; cefoxitin, ≥ 32 μg mL**^–^**^1^; ceftiofur, ≥ 8 μg mL**^–^**^1^; ceftriaxone, ≥ 4 μg mL**^–^**^1^; chloramphenicol, ≥ 32 μg mL**^–^**^1^; ciprofloxacin, ≥1 μg mL**^–^**^1^; gentamicin, ≥16 μg mL**^–^**^1^; nalidixic acid, ≥32 μg mL**^–^**^1^; streptomycin, ≥32 μg mL**^–^**^1^; sulfisoxazole, ≥256 μg mL**^–^**^1^; tetracycline, ≥16 μg mL**^–^**^1^; and trimethoprim/sulfamethoxazole, ≥4/76 μg mL**^–^**^1^. Since the CMV3AGNF plate has maximum concentrations of 16 and 256 μg mL**^–^**^1^ for azithromycin and sulfisoxazole, we could not evaluate the NARMS resistance breakpoints of ≥32 and ≥512 μg mL**^–^**^1^, respectively. For enterococci, the panel of 16 antimicrobials and breakpoints for classification as resistant were as follows: chloramphenicol, ≥32 μg mL**^–^**^1^; ciprofloxacin, ≥4 μg mL**^–^**^1^; daptomycin, ≥8 μg mL**^–^**^1^; erythromycin ≥ 8 μg mL**^–^**^1^; gentamicin, ≥512 μg mL**^–^**^1^; kanamycin, ≥1,024 μg mL**^–^**^1^; lincomycin, ≥8 μg mL**^–^**^1^; linezolid, ≥8 μg mL**^–^**^1^; nitrofurantoin, ≥64 μg mL**^–^**^1^; penicillin, ≥16 μg mL**^–^**^1^; quinupristin/dalfopristin, ≥4 μg mL**^–^**^1^; streptomycin, ≥1,024 μg mL**^–^**^1^; tetracycline, ≥16 μg mL**^–^**^1^; tigecycline, ≥0.5 μg mL**^–^**^1^; tylosin, ≥32 μg mL**^–^**^1^; and vancomycin, ≥32 μg mL**^–^**^1^. The CMV3AGPF plate has a maximum concentration of 64 μg mL**^–^**^1^ for nitrofurantoin, thus we could not evaluate the NARMS resistance breakpoint of ≥128 μg mL**^–^**^1^.

## Results

### Enumeration of Fecal Indicator Bacteria

**FIGURE 1:**
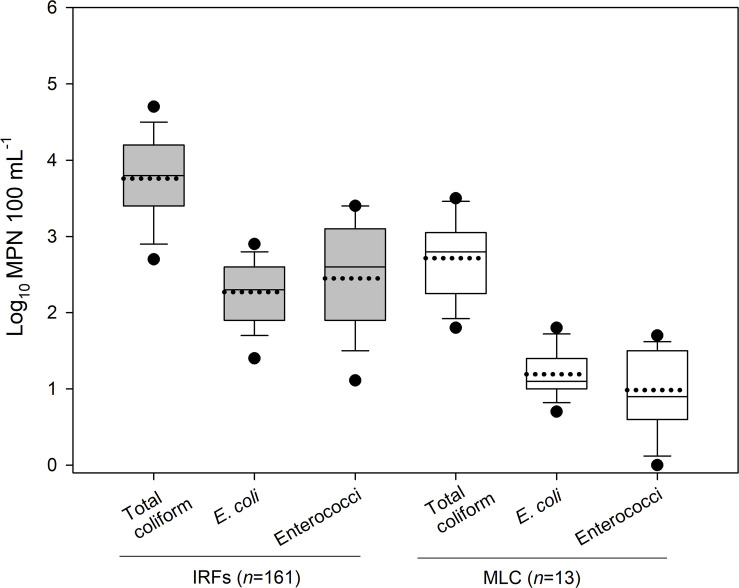
The level (log_10_ MPN 100 mL**^–^**^1^) of total coliform, *E. coli*, and enterococci in the irrigation return flows (IRFs) and background canal site (MLC) in the Upper Snake Rock watershed in south-central Idaho. The black horizontal lines in the box plots, from bottom to top including the whisker caps, represent the 10th, 25th, 50th, 75th, and 90th percentiles, while the black dotted line is the mean and black circles are the 5th and 95th percentiles.

In 2018, 161 and 13 water samples were collected from the IRFs and background MLC site between 9 January and 18 Dec, respectively. In Figure 1, the MPN results for total coliforms, *E. coli*, and enterococci are presented as box plots. In the IRF samples, the respective mean levels were log_10_ 3.8, 2.3, and 2.5 MPN 100 mL**^–^**^1^, while in MLC samples the mean levels were lower at log_10_ 2.7, 1.2, and 1.0 MPN 100 mL**^–^**^1^. Table 1 shows the minimum, maximum, and mean levels for the FIB at each of the sampling sites, with 1 CFU 100 mL**^–^**^1^ being the lowest level detected during the entire study. The levels for total coliforms, *E. coli*, and enterococci in the IRFs ranged from log_10_ 1.9 to 4.9, 1.0 to 3.4, and 0 to 3.7 MPN 100 mL**^–^**^1^, respectively. At the MLC site, the respective ranges were log_10_ 1.8 to 3.5, 0.7 to 1.8, and 0 to 1.7 MPN mL**^–^**^1^. The sampling sites with the greatest mean levels of total coliforms and *E. coli* were NC and DC at log_10_ 4.4 and 2.9 MPN 100 mL**^–^**^1^, respectively, while NC, TFC, and HC had the greatest mean levels of enterococci at log_10_ 3.1 MPN 100 mL**^–^**^1^. For all FIB, mean levels at each of the IRF sites were determined to be statistically greater (*P* < 0.05) than mean levels at MLC.

**TABLE 1:** Summary of total coliform, *E. coli*, and enterococci levels in irrigation return waters in the Upper Snake Rock watershed in south-central Idaho.

		**Log_10_MPN 100 mL^–1^**		
**Site**		**Total coliforms**	***E*. *coli***	**Enterococci**
NC	Min	2.8	1.7	1.9
	Max	4.9	2.8	3.7
	Mean	4.4	2.3	3.1
DC	Min	1.9	1.8	0.0
	Max	4.2	3.4	3.4
	Mean	3.7	2.9	2.6
MC	Min	3.4	1.4	1.5
	Max	4.5	2.8	3.4
	Mean	4.1	2.3	2.8
IC	Min	2.5	1.6	1.4
	Max	4.8	2.9	3.5
	Mean	4.1	2.4	2.9
CD	Min	2.6	1.3	0.8
	Max	4.5	3.2	3.6
	Mean	4.0	2.4	2.8
RCP	Min	2.7	1.0	1.4
	Max	4.4	2.7	3.5
	Mean	3.9	2.2	2.8
TFC	Min	3.2	1.1	1.8
	Max	4.7	2.8	3.7
	Mean	4.3	2.5	3.1
HC	Min	2.9	1.9	1.9
	Max	4.7	2.9	3.4
	Mean	4.1	2.6	3.1
MLC	Min	1.8	0.7	0.0
	Max	3.5	1.8	1.7
	Mean	3.0	1.3	1.2

**FIGURE 2:**
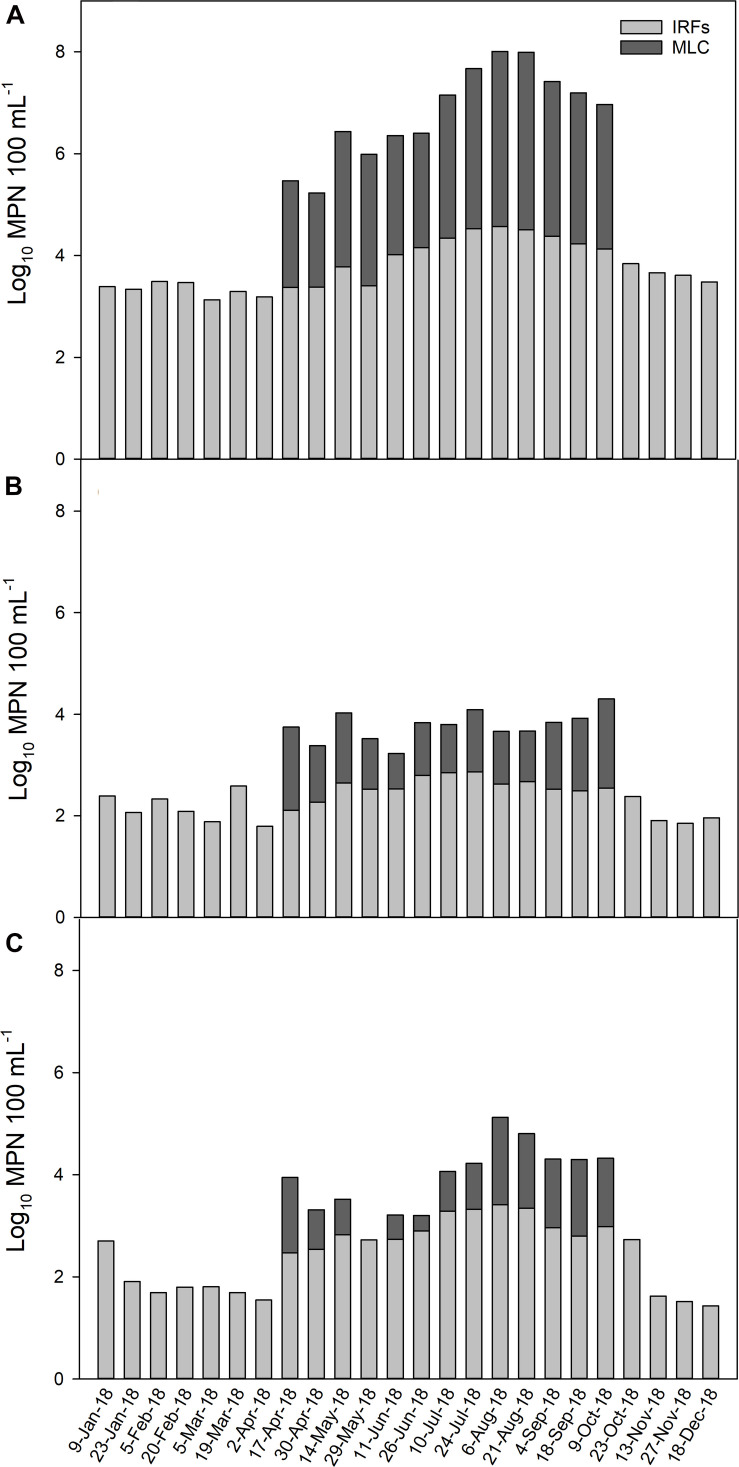
The average level (log_10_ MPN 100 mL**^–^**^1^) of **(A)** total coliform, **(B)** *E. coli*, and **(C)** enterococci in the irrigation return flows (IRFs) and background canal site (MLC) on each sampling day from January to December 2018. Snake River water was diverted into MLC from 10 April to 21 October, 2018.

To illustrate how the MPN levels changed throughout the year across all IRFs and at MLC, the mean MPN 100 mL**^–^**^1^ for the FIB on each collection day (total of 26 d) is presented in Figure 2. Total coliform levels were lower at approximately log_10_ 3 MPN 100 mL**^–^**^1^ from January through May, then on the first sampling date in June the levels began to increase above log_10_ 4 MPN 100 mL**^–^**^1^ until the greatest mean level of log_10_ 4.6 MPN 100 mL**^–^**^1^ was noted on 6 August, followed by a gradual decline thereafter (Figure 2A). In the MLC samples, total coliform levels from 17 April to 9 October were, on average, 1.5-fold lower than IFR levels. *Escherichia coli* mean levels in the IRFs tended to fluctuate between log_10_ 2 and 3 MPN 100 mL**^–^**^1^ throughout much of the year, with the highest levels generally occurring during summer and early fall (Figure 2B). The *E. coli* levels in MLC samples were 2.3-fold lower on average than in IFR samples. Enterococci mean levels were approximately log_10_ 2 MPN 100 mL**^–^**^1^ from 23 January to 2 April, then increased on 17 April and reached a maximum level of log_10_ 3.4 MPN 100 mL**^–^**^1^ on 6 August and then decreased to background levels on 13 November (Figure 2C). In MLC samples, enterococci levels were 3.5-fold lower on average than in IRF samples.

### Characterization of *Escherichia coli* and Enterococcal Isolates

A total of 806 *E. coli* and 605 enterococcal isolates were obtained from the water samples, but only 187 and 185 isolates were selected for antimicrobial susceptibility testing. More specifically, 171 and 16 *E. coli* and 173 and 12 enterococci were chosen from the IRFs and MLC samples, respectively. Phylotyping and sequencing results for *E. coli* and enterococci, respectively, were used to select a representative subset of isolates from each sampling site and water collection event. Phylotyping of *E. coli* revealed that all seven phylogroups were represented at almost all of the sampling sites, except for three sites lacking the following phylogroups: A1 at DC, B2.2 at RCP, and D2 at IC (Table 2). Across all sampling sites, phylotype B1 (47/187; 25%) was the most abundant, while D2 (14/187; 8%) was the least abundant. At MLC, phylotype B1 was also the most abundant (4/16; 25%).

**TABLE 2:** Phylogroups of *E. coli* isolates (*n* = 187) from the irrigation return flows and background canal site (MLC).

**Site**	**Phylogentic group**						
	**AO**	**Al**	**Bl**	**B2.2**	**B2.3**	**Dl**	**D2**
NC	2	2	4	2	2	1	2
DC	2	0	6	2	2	6	1
MC	2	5	6	1	4	6	1
IC	2	4	6	1	3	4	0
CD	4	2	7	1	6	6	3
RCP	6	4	6	0	6	4	2
TFC	3	1	4	5	1	1	2
HC	3	1	4	4	1	3	2
MLC	1	3	4	2	2	3	1
Σ=	25	22	47	18	27	34	14

Based on 16S rRNA gene sequencing, the enterococcal isolates were determined to be most closely aligned with the following *Enterococcus* species: *E. casseliflavus*, *E. faecalis*, *E. faecium*, *E. hirae*, *E. malodoratus*, *E. mundtii*, *E. phoeniculicola*, and *E. thailandicus* (Table 3). The species that were most prevalent were *E. casseliflavus* (38/185; 21%), *E. faecalis* (37/185; 20%), *E. faecium* (55/185; 30%) and *E. mundtii* (40/185; 22%), which were detected at all sampling sites with occurrences ranging from 1 to 10 per site. *Enterococcus hirae*, *E. malodoratus*, *E. phoeniculicola*, and *E. thailandicus* only represented 8% (15/185) of all enterococcal isolates and they were detected at some of the IFR sampling sites, but not at MLC.

**TABLE 3:** The number of *Enterococcus* spp. isolated from the irrigation return flows and background canal site (MLC) that v/ere characterized for antibiotic resistance in the present study.

**Site**	***n***	**No. of isolates**							
		***E. casseliflavus***	***E. faecalis***	***E. faecium***	***E. hirae***	***E. malodoratus***	***E. mundtii***	***E. phoeniculicola***	***E*. *thailandicus***
NC	13	3	3	4	0	0	2	0	1
DC	23	5	5	4	3	0	4	1	1
MC	26	6	8	7	1	0	4	0	0
IC	26	5	8	7	1	0	5	0	0
CD	28	7	4	8	0	0	7	1	1
RCP	26	4	2	10	1	1	6	0	2
TFC	15	3	3	4	0	0	5	0	0
HC	16	2	3	4	1	0	6	0	0
MLC	12	3	1	7	0	0	1	0	0
Σ=	185	38	37	55	7	1	40	2	5

### Antimicrobial Resistance of *Escherichia coli*

*Escherichia coli* isolates from the IRFs and MLC were resistant to 12 of 14 antimicrobials (Table 4). For IRF isolates, resistance to tetracycline (21/171; 12%) was the most commonly encountered, followed by ampicillin (13/171; 8%), sulfisoxazole (12/171; 7%), amoxicillin/clavulanic acid (10/171; 6%), and cefoxitin (8/171; 5%). Overall resistance to azithromycin, ceftiofur, chloramphenicol, ciprofloxacin, nalidixic acid, streptomycin, and trimethoprim/sulfamethoxazole was 9.4% of the IRF *E. coli* isolates, with none being resistant to gentamicin. The *E. coli* isolates from MLC were resistant to all but azithromycin and gentamicin, with resistance to nalidixic acid and sulfisoxazole (both 5/16; 31%) being the most common, while resistance to amoxicillin/clavulanic acid, cefoxitin, ceftiofur, and ceftriaxone (all 2/16; 13%) being the least common.

**TABLE 4:** Antimicrobial resistance of *E. coli* isolates from the irrigation return flows (IRFs) and background canal site (MLC).

**Antimicrobial**	**Breakpoint (μg mL**^–^**^1^)**	**No. of resistant (%)**	
		**IRFs**	**MLC**
		**(*n* = 171)**	**(*n* = 16)**
Amoxicillin/clavulanic acid	≥32/16	10 (5.8)	2 (12.5)
Ampicillin	≥32	13 (7.6)	3 (18.8)
Azithromycin	≥16	1 (0.6)	0 (0)
Cefoxitin	≥32	8 (4.7)	2 (12.5)
Ceftiofur	≥8	1 (0.6)	2 (12.5)
Ceftriaxone	≥4	2 (1.2)	2 (12.5)
Chloramphenicol	≥32	4 (2.3)	3 (18.8)
Ciprofloxacin	≥1	4 (2.3)	5 (31.3)
Gentamicin	≥16	0 (0)	0 (0)
Nalidixic Acid	≥32	4 (2.3)	5 (31.3)
Streptomycin	≥32	9 (5.3)	3 (18.8)
Sulfisoxazole	≥256	12 (7)	5 (31.3)
Tetracycline	≥16	21 (12.3)	4 (25)
Trimethoprim/sulfamethoxazole	≥4/76	2 (1.2)	4 (25)

Twenty-five resistance patterns were detected in the *E. coli* isolates (Table 5). None of the *E. coli* were pan-resistant, but 7% (12/171) from the IRFs and 25% (4/16) from MLC were MDR. A total of nine MDR patterns with resistance to three and five antimicrobials were observed in *E. coli* from the IRFs. The other resistant IRF *E. coli*, however, were only resistant to 1 (13/171; 8%) and 2 (13/171; 8%) different classes of antimicrobials. The most common resistance patterns among the IRF *E. coli* were Tet and Aug2AmpFox. In *E. coli* from MLC, three MDR patterns were observed with four isolates being resistant to 5, 7, or 10 antimicrobials. In the latter case, one isolate was resistant to AmpXnlAxoChlCipNalStrFisTetSxt, which consists of seven antimicrobial drug classes. Seventy-eight percent (133/171) and 50% (8/16) of the IRF and MLC isolates were pan-susceptible, respectively.

**TABLE 5:** Single and multidrug resistance patterns in *E. coli* from the irrigation return flows (IRFs) and background canal site (MLC).

**Resistance pattern**	**No. of antimicrobials**	**No. of drug classes**	**No. of isolates**	
			**IRFs**	**MLC**
			**(*n* = 171)**	**(*n* = 16)**
Pan-susceptible	0	0	133	8
Azi	1	1	1	0
Fis	1	1	1	1
Fox	1	1	1	0
Str	1	1	1	0
Tet	1	1	9	0
AmpTet	2	2	1	0
Aug2Fis	2	2	1	0
ChlTet	2	2	1	0
CipNal	2	2	1	1
StrTet	2	2	1	0
XnlNal	2	2	1	0
AmpStrTet	3	3*	1	0
AxoCipFis	3	3*	1	0
Aug2AmpFox	3	2	7	1
Aug2FisTet	3	3*	2	0
CipTetStr	3	3*	1	0
FisTetStr	3	3*	1	0
AmpNalFisSxt	4	3*	1	0
AxoCipNalFis	4	3*	1	0
FisStrSxtTet	4	3*	1	0
AmpChlStrFisTet	5	5*	3	0
Aug2AmpAxoFo Xnl	5	2	0	1
CipNalFisTetSxt	5	3*	0	1
ChlCipNalStrFis TetSxt	7	5*	0	2
AmpXnIAxoChlCip NalStrFisTetSxt	10	7*	0	1

Aug2, amoxicillin/clavulanic acid; Amp, ampicillin; Azi, azithromycin; Fox, cefoxitin; XnL. ceftiofur; Axo, ceftriaxone; Chl, chloramphenicol; Cip, ciprofloxacin; Gen, gentamicin; Nal, nalidixic acid; Str, streptomycin; Fis, sulfisoxazole; Tet, tetracyline; Sxt, trimethoprim/sulfamethoxazole; Pan-susceptible, susceptible to all antimicrobials tested.

*Denotes multidrug resistance based on resistance to antimicrobials in three or more drug classes.

The distribution of the 38 IRF and eight MLC resistant *E. coli* isolates by phylogroup and resistance pattern is presented in Table 6. All seven phylogroups were represented in isolates from the IRFs, while five out of seven were represented in the MLC isolates. In IRF isolates, B1 (eight isolates) and D1/D2 (three isolates each) were the most and least abundant groups, respectively. In MLC isolates, the phylogroups represented were A1 (three isolates), B1 (one isolate), B2.3 (two isolates), D1 (one isolate), and D2 (one isolate). Among the IRF isolates, the greatest number of resistances was to five antimicrobials, and was noted in isolates belonging to groups A1 and D1, while an MLC isolate belonging to group A1 was resistant to 10 antimicrobials. Two other MLC isolates belonging to groups A1 and B2.3 were also found to be resistant to the same seven antimicrobials (i.e., ChlCipNalStrFisTetSxt).

**TABLE 6:** The distribution of resistant *E. coli* isolates from the irrigation return flows (IRFs) and background canal site (MLC) by phylogroup and resistance pattern.

**Source**	**No. of isolates**	**Phylogenetic group**	**Resistance pattern**
IRFs	1	A0	Fis
	1	A0	Tet
	1	A0	AxoCipFis
	1	A0	AmpStrTet
	1	A0	Aug2AmpFox
	1	A0	AxoCipNalFis
	3	A1	Tet
	1	A1	Aug2Fis
	1	A1	StrTet
	2	A1	AmpChlStrFisTet
	1	B1	Azi
	1	B1	Tet
	1	B1	AmpTet
	1	B1	ChlTet
	1	B1	XnlNal
	1	B1	Aug2AmpFox
	1	B1	Aug2AmpFox
	1	B1	FisTetStr
	1	B2.2	Fox
	1	B2.2	Tet
	2	B2.2	Aug2AmpFox
	1	B2.2	CipTetStr
	1	B2.3	Str
	1	B2.3	Tet
	1	B2.3	CipNal
	1	B2.3	Aug2AmpFox
	2	B2.3	Aug2FisTet
	1	D1	Tet
	1	D1	AmpNalFisSxt
	1	D1	AmpChlStrFisTet
	1	D2	Tet
	1	D2	Aug2AmpFox
	1	D2	FisStrSxtTet
MLC	1	A1	Aug2AmpFox
	1	A1	ChlCipNalStrFisTetSxt
	1	A1	AmpXnIAxoChlCipNalStrFisTetSxt
	1	B1	CipNal
	1	B2.3	ChlCipNalStrFisTetSxt
	1	B2.3	Fis
	1	D1	Aug2AmpAxo FoxXnl
	1	D2	CipNalFisTetSxt

### Antimicrobial Resistance of Enterococci

*Enterococcus* spp. from the IRFs were resistant to 10 of 16 antimicrobials (Table 7), with none being resistant to gentamicin, linezolid, tigecycline, and vancomycin. Resistance to lincomycin (130/173; 75%) was the most prevalent, followed by nitrofurantoin (50/173; 29%) and tetracycline (20/173; 12%). Resistance to chloramphenicol, ciprofloxacin, daptomycin, erythromycin, kanamycin, penicillin, streptomycin, and tylosin tartrate was relatively uncommon at only 8% of the IRF enterococcal isolates. Enterococcal isolates from MLC were only resistant to four of 16 antimicrobials, specifically lincomycin (8/12; 67%), nitrofurantoin (6/12; 50%), quinupristin/dalfopristin (1/12; 8%), and tetracycline (2/12; 17%).

**TABLE 7:** Antimicrobial resistance of enterococcal isolates from the irrigation return flows (IRFs) and background canal site (MLC).

**Antimicrobial**	**Breakpoint (μ g mL^–1^)**	**No. of resistant (%)**	
		**IRFs**	**MLC**
		**(*n* = 173)**	**(*n* = 12)**
Chloramphenicol	≥32	2 (1.2)	0 (0)
Ciprofloxacin	≥4	2 (1.2)	0 (0)
Daptomycin	≥8	1 (0.6)	0 (0)
Erythromycin	≥8	1 (0.6)	0 (0)
Gentamicin	≥512	0 (0)	0 (0)
Kanamycin	≥1024	2 (1.2)	0 (0)
Lincomycin	≥8	130 (75.1)	8 (66.7)
Linezolid	≥8	0 (0)	0 (0)
Nitrofurantoin	≥64	50 (28.9)	6 (50)
Penicillin	≥16	1 (0.6)	0 (0)
Quinupristin/dalfopristin	≥4	33 (19.1)	1 (8.3)
Streptomycin	≥1024	2 (1.2)	0 (0)
Tetracycline	≥16	20 (11.6)	2 (16.7)
Tigecycline	≥0.5	0 (0)	0 (0)
Tylosin tartrate	≥32	2 (1.2)	0 (0)
Vancomycin	≥32	0 (0)	0 (0)

Seventeen resistance patterns were detected in the enterococcal isolates across all species (Table 8). In IRF enterococcal isolates, the most common resistance patterns were Lin (71/173; 41%), LinSyn (30/173; 17%), Nit (18/173; 10%), and LinNit (15/173; 9%). A total of six MDR patterns were observed with 12 isolates being resistant to 3, 4, or 6 antimicrobial drug classes, with LinNitTet (5/173; 3%) as the most abundant MDR pattern. In MLC isolates, Lin (4/12; 33%) and Nit (3/12; 25%) were the most common drug resistances, while LinNetTet (2/12; 17%) was the only MDR pattern. Only 9% (15/173) and 8% (1/12) of the IRF and MLC isolates were pan-susceptible, respectively.

**TABLE 8:** Single and multidrug resistance patterns in enterococcal isolates from the irrigation return flows (IRFs) and background canal site (MLC).

**Resistance pattern**	**No. of antimicrobials**	**No. of drug classes**	**No. of isolates**	
			**IRFs (*n* = 173)**	**MLC (*n* = 12)**
Pan-susceptible	0	0	15	1
Lin	1	1	71	4
Nit	1	1	18	3
Tet	1	1	1	0
ChlLin	2	2	1	0
DapLin	2	2	1	0
LinNit	2	2	15	1
LinSyn	2	2	30	1
LinTet	2	2	1	0
LinTylt	2	2	1	0
NitSyn	2	2	1	0
NitTet	2	2	7	0
KanNitTet	3	3*	1	0
LinNitTet	3	3*	5	2
ChlLinSynTet	4	4*	1	0
CipLinNitTet	4	4*	2	0
LinNitPenStrTet	5	4*	1	0
EryKanLinStrSynTetTylt	7	6*	1	0

Chl, chloramphenicol; Cip, ciprofloxacin; Dap, daptomycin; Ery, erythromycin; Kan, kanamycin; Lin, lincomycin; Lzd, linezolid; Nit, nitrofurantoin, Pen, penicillin; Str, streptomycin; Syn, quinupristin/dalfopristin; Tet, tetracyline; Tylt, tylosin tartrate; Pan-susceptible, susceptible to all antimicrobials tested.

*Denotes multidrug resistance based on resistance to antimicrobials in three or more drug classes.

To better understand AMR patterns among the enterococcal isolates, the results were broken down according to species (Table 9). Only one isolate from an IRF was identified as *E*. *malodoratus* and it was pan-susceptible. Very few resistant isolates were identified as *E. hirae* (five Lin, one LinSyn), *E. phoeniculicola* (two Lin), and *E. thailandicus* (three LinNit, one LinTet, and one LinNitTet). The majority of the *Enterococcus* spp. were resistant to Lin (138/185; 75%). The species with the greatest to smallest percentage of lincomycin-resistant isolates were *E. thailandicus* (5/5; 100%), *E. phoeniculicola* (2/2; 100%), *E. mundtii* (35/40; 88%), *E. faecalis* (33/37; 89%), *E. hirae* (6/7; 86%), *E. casseliflavus* (30/38; 79%), and *E. faecium* (27/55; 49%). The second most abundant resistance pattern was Nit (56/185; 30%) and 75% (42/56) of the isolates were identified as *E. faecium*. Overall, 13 enterococcal isolates, predominantly *E. faecalis*, *E. faecium*, *E. casseliflavus*, and *E. thailandicus*, were determined to be MDR to up to six different drug classes. MDR was more common among *E. faecium* than the other enterococcal species; eight isolates had MDR patterns of LitNetTet (five isolates), CipLinNitTet (two isolates), KanNitTet (one isolate), and LinNitPenStrTet (one isolate).

**TABLE 9:** Resistance patterns of *Enterococcus* spp. from the irrigation return flows and background canal site.

**Resistance pattern**	**No. of isolates**							
	***E. casseliflavus* (*n* = 38)**	***E*. *faecalis* (*n* = 37)**	***E. faecium* (*n* = 55)**	***E. hirae* (*n* = 7)**	***E. malodoratus* (*n* = 1)**	***E. mundtii* (*n* = 40)**	***E. phoeniculicola* (*n* = 2)**	***E*. *thailandicus* (*n* = 5)**
Lin	24	3	9	5	0	32	2	0
Nit	0	2	17	0	0	2	0	0
Tet	1	0	0	0	0	0	0	0
ChlLin	1	0	0	0	0	0	0	0
DapLin	0	0	0	0	0	1	0	0
LinNit	2	0	9	0	0	2	0	3
LinSyn	1	28	1	1	0	0	0	0
LinTet	0	0	0	0	0	0	0	1
LinTylt	1	0	0	0	0	0	0	0
NitSyn	0	1	0	0	0	0	0	0
NitTet	0	0	7	0	0	0	0	0
KanNitTet	0	0	1	0	0	0	0	0
LinNitTet	1	0	5	0	0	0	0	1
ChlLinSynTet	0	1	0	0	0	0	0	0
CipLinNitTet	0	0	2	0	0	0	0	0
LinNitPenStrTet	0	0	1	0	0	0	0	0
EryKanLinStrSynTetTylt	0	1	0	0	0	0	0	0

*Chl, chloramphenicol; Cip, ciprofloxacin; Dap, daptomycin; Ery, erythromycin; Kan, kanamycin; Lin, lincomycin; Lzd, linezolid; Nit, nitrofurantoin; Pen, penicillin; Str, streptomycin; Syn, quinupristin/dalfopristin; Tet, tetracyline; Tylt, tylosin tartrate.*

## Discussion

The purpose of this study was to characterize resistance to one or more antimicrobials in *E. coli* and enterococcal isolates obtained from IFRs and a background canal site in the USR watershed. This mixed-use watershed is located in a high-desert region that supports intensive agricultural production, with a large portion utilized for cash crops, forages and dairy production, but it is also populated with small towns and cities, as well as an extensive rural population where households use on-site underground septic systems for wastewater treatment. Land application of manure, compost, and wastewater from dairy operations is a common practice, which can improve soil quality and provide nutrients for crops (Dungan et al., 2011, 2017a). The USR watershed presents a unique opportunity to study waterborne contaminants because rainfall is limited, and thus the vast majority of water that is cycled within the watershed is a direct result of crop irrigation during the growing season (Bjorneberg et al., 2020). Previous research in the watershed has shown that antimicrobial residue and ARG levels in IFRs were slightly elevated compared with the incoming surface water from the Snake River that is diverted into canals, laterals, and ditches (Dungan et al., 2017b; Dungan and Bjorneberg, 2020).

*Escherichia coli* and enterococci are important indicators for understanding the impact of fecal pollution on water resources, but reports on AMR among these organisms in IRFs are lacking. On average, the FIB levels in the IRFs were found to be greater than in water samples from the background site, which can be expected since the irrigation returns receive water from surface and subsurface drainage (Bjorneberg et al., 2008). The influence of irrigation on the FIB levels in the IRFs was evident as the levels increased when the irrigation season commenced in mid-April and stayed elevated until irrigation ceased in mid-October. An earlier Investigation of a rangeland watershed in southwest Idaho suggested that IRFs were responsible for flushing total and fecal coliforms from some fields into a stream during the irrigation season (Stephenson and Street, 1978). While FIB are likely carried into IRFs in surface and subsurface drainage from a variety of sources (e.g., fields, septic systems, urban drainage), it is also possible that the increased flow/turbulence in the irrigation returns during the irrigation season resuspends FIB that reside in sediments. As a result, the irrigation return FIB could be derived from fecal contamination or naturalized extraintestinal populations that populate soils and sediments (Bradshaw et al., 2016). Using AMR analysis of fecal coliforms in water samples from a mixed-use watershed in Georgia, human sources were determined to contribute a majority of the baseflow isolates in urbanized areas, while livestock sources were responsible for the majority of the baseflow isolates in rural areas (Burnes, 2003).

Phylotyping was performed on *E. coli* to assess the diversity among the isolates, with a large proportion (92/187; 49%) belonging to group B and a nearly equal split between groups A (47/187; 25%) and D (48/187; 26%). Of the seven phylogroups that were encountered, B1 (47/187; 25%) was the most abundant, while the least abundant was D2 (14/187; 7%). The existence of distinct phylogroups within *E. coli* has long been recognized and phylogroup determination is a useful characterization tool since a strain’s ecological niche and pathogenicity vary with its phylogenetic origins (Gordon et al., 2008; Clermont et al., 2015). Many studies have shown that isolates responsible for extraintestinal disease in humans belong mainly to group B2 and to a lesser extent group D (Picard et al., 1999; Tenaillon et al., 2010). In contrast, Escobar-Paramo et al. (2004a) found that *E. coli* responsible for acute and severe diarrhea were not found in groups B2 and D, while those causing chronic and mild diarrhea were distributed among all phylogroups. In surface water samples and animal feces collected in the Upper Midwest of the United States, 57 and 51% of *E. coli* isolates were found to belong to group B1, respectively, with substantially fewer isolates belonging to all other phylogroups (Johnson et al., 2017). However, they reported that about 95% of the strains among water and fecal isolates were non-extraintestinal, with those determined to be extraintestinal to be predominantly from group B2. In humans, group A strains were predominant (41%) [followed by group B2 (26%), then B1 and D (17% each)], while in animals, group B1 strains were predominant (41%) [followed by group A (22%), B2 (21%), and D (16%)] (Tenaillon et al., 2010). In a study of 300 *E. coli* isolates from a variety of animal production systems across the United States, all seven of the phylogroups were represented with phylogroups B1 (107 isolates or 36%) and B2.2 (7 isolates or 2.3%) being the most and least abundant, respectively (Ducey et al., 2020). Given the distribution of the *E. coli* strains among the phylogroups in the present study with B1 being the most abundant group (i.e., 47/187; 25%), the evidence would suggest that many have originated from animals.

Although commensal bacteria are generally considered harmless, they can act as a reservoir of many ARGs, which may be organized within genetic elements such as integrons (Bailey et al., 2010; Fard et al., 2011; Lebreton et al., 2013). A growing body of evidence indicates that ARGs can readily be transferred among microbial species (including between commensals and pathogens) mainly *via* transformation and conjugation events (Marshall et al., 2009; Stokes and Gillings, 2011). It is not known if the *E. coli* and enterococcal isolates from the present study are pathogenic to humans and other animals; however, none of the *E. coli* were found to contain genes encoding for intimin and Shiga toxins, thus confirming that they were not enterohemorrhagic *E. coli* (data not shown) (Fagan et al., 1999; Sharma and Dean-Nystrom, 2003). The present data can be of value to determine the possible spread of these bacteria and their ARGs to the Snake River, which is a recreational waterbody and the 9th longest river in the United States. Because water can move substantial distances in rivers, waterways could be a dominant route by which ARGs are disseminated throughout the environment (Pruden et al., 2012; Keen et al., 2018).

From the susceptibility testing results for *E. coli*, 75% of the 187 isolates were pan-susceptible, while 16% were resistant to antimicrobials from one or two drug classes and 9% were MDR. When just considering the number of resistant isolates per individual antimicrobial, tetracycline resistance was the most prevalent (13%), followed by sulfisoxazole (9%) and ampicillin (9%) resistance. The high rate of tetracycline resistance is not surprising given that tetracycline resistance genes [e.g., *tet*(B), *tet*(M), and *tet*(X)] have been detected in IRF and MLC samples (Dungan and Bjorneberg, 2020). While we have not detected tetracycline and ampicillin residues, trace quantities of certain sulfonamides have been detected (Dungan et al., 2017b). Tetracycline resistance genes and other ARGs are generally more abundant in riverine environments impacted by urban and agricultural activities (Pei et al., 2006; Storteboom et al., 2010). Keen et al. (2018) found that tetracycline resistance genes were more abundant in the Sumas River agricultural watershed of British Columbia than in a forested headwater control site. It was speculated that higher intensity rainfall events, agricultural activities, and land use practices contributed to elevated gene levels in the watershed mainly as a result of soil erosion. Many ARGs are present in native soils, but the abundance of the genes is dramatically greater in cropland soils, especially those that have a history of being treated with animal manures and biosolids (Knapp et al., 2010; Dungan et al., 2019). Fecal coliforms from cattle, humans, and other domesticated animals and wildlife are known to be resistant to a variety of antimicrobials and their resistance patterns have been used for source tracking in watersheds (Whitlock et al., 2002; Burnes, 2003; Ducey et al., 2020).

Multidrug resistance was observed among the *E. coli* from both the IRFs and MLC. Twelve MDR patterns with resistance to up to seven drug classes (10 antimicrobials) was noted in 17 isolates that were found among all of the phylogroups. Similarly, Cho et al. (2018) found that a small percentage of *E. coli* isolates (15/496; 3%) were MDR in surface waters from the Upper Oconee watershed, which is a mixed-used watershed in northern Georgia. Eleven totally different MDR patterns were detected, including one isolate that was resistant to seven antimicrobials (i.e., AmpCipNalStrFisTetSxt) and belonged to phylogroup B1. In a survey of *E. coli* isolates from surface water in the Grand River watershed in Waterloo, Canada, 17 out of 93 isolates (18%) were found to be resistant to 2–5 antimicrobial classes, but the MDR patterns were not provided (Kadykalo et al., 2020). In the Cho et al. (2018) and Kadykalo et al. (2020) studies, it should be noted that the researchers used the CMV3AGNF Sensititre plate as used in the present study. In a study of *E. coli* from aquatic environments in Rio de Janeiro, Brazil, 66 out of 178 isolates (37%) were resistant to at least one of 11 antimicrobials tested and 17 isolates were MDR (de Luca Rebello and Regua-Mangia, 2014). The lowest percentage of MDR *E. coli* were recovered from agricultural wastewaters (4%) with higher percentages in recreational waters (13%) and residential (8%), industrial (11%), and hospital (17%) wastewaters (de Luca Rebello and Regua-Mangia, 2014). Similarly, MDR prevalence among *E. coli* from the Seine river watershed in France was lowest in agricultural (8%) and forest (1%) non-point sources and higher in municipal wastewaters (34%), rivers (35%), and hospital wastewaters (65%) (Servais and Passerat, 2009).

Surface waters contain a wide variety of enterococcal species, which are influenced by anthropogenic activities in the surrounding environment and wild/domesticated animals (Messi et al., 2006; Meinersmann et al., 2008; Furtula et al., 2013). Commonly isolated enterococcal species from surface waters are *E. faecalis* and *E. faecium*, as well as *E. casseliflavus*, *E. gallinarum*, *E. durans*, *E. hirae*, and *E. mundtii* (Švec and Sedláèek, 1999; Łuczkiewicz et al., 2010). In contrast to the *E. coli* isolates, the majority of the enterococcal isolates in the present study were found to be AMR and the single highest resistance was to lincomycin. In surface water samples collected in an area with intensive poultry production, the highest level of resistance among 36 enterococcal isolates was to lincomycin (88%), followed by tetracycline (24%) (Furtula et al., 2013). Although lincomycin is commonly used in poultry production, we have not previously detected lincomycin residues in the USR watershed (Dungan et al., 2017b), which is not surprising given that it is not approved for use in dairy cattle in the United States. Jackson et al. (2011) showed that the most common AMR phenotype among enterococcal isolates from dairy cattle was to lincomycin (587/636; 92%). In addition, they detected at least 10 enterococcal species and lincomycin-resistant isolates were abundant among each of the different species Jackson et al. (2011). Similarly, 10 different enterococcal species were detected in surface water samples from the Upper Oconee watershed and the majority (564/637; 89%) were resistant to lincomycin (Cho et al., 2020a). In the present study, seven out of eight enterococcal species (except *E*. *malodoratus*) were resistant to lincomycin (range of 49–100% isolates per species). The high rate of lincomycin resistance among enterococci is likely not a result of exposure to the antimicrobial, but due to the fact that most enterococci, with the exception of *E. durans*, are intrinsically resistant to lincomycin (Gilmore et al., 2002).

While the majority of the enterococcal isolates in the present study were AMR, 7% (13/185) were MDR to as many as six drug classes with six different patterns, and these MDR isolates were only found among four of eight species: *E. casseliflavus*, *E. faecalis*, *E. faecium*, and *E. thailandicus*. Similarly, Cho et al. (2020a) found that 8% (51/637) of enterococcal isolates from an agricultural watershed were MDR to as many as five drug classes with 18 different patterns; six of nine species were found to be MDR, including *E. casseliflavus*, *E. faecalis*, *E. faecium*, *E. hirae*, *E. gallinarum*, and *E. mundtii*, and no MDR was detected among *E. avium*, *E. durans*, and *E. pallens*. The differences between the results of Cho et al. (2020a) and the present study are likely related to a number of factors such as watershed characteristics, regional climates, and *Enterococcus* spp. source. *Enterococcus faecalis* and *faecium* are commensals from the gastrointestinal tract of warm-blood animals, but can cause life-threating infections in humans and account for about 80–90% and 5–15% of all clinical isolates, respectively (Cetinkaya et al., 2000). *Enterococcus hirae* and *durans* are infrequently isolated from human clinical samples and are known pathogenic agents in young animals (Gilmore et al., 2002). *Enterococcus casseliflavus* and *gallinarum* are intrinsically resistant to low levels of vancomycin (Dutka-Malen et al., 1994) and are also rarely isolated in clinical samples (Reid et al., 2001), but they are detected in horse, cattle, and bird feces (Thal et al., 1995; Haenni et al., 2009) and cause urinary tract infections in canines (Simjee et al., 2002). Given that the AMR enterococcal isolates in the present study could potentially cause disease in exposed humans, an additional concern is that therapeutic treatments could fail. All of the AMR enterococci were resistant to antimicrobials that are deemed “important,” “highly important,” or “critically important” for human medicine by the World Health Organization (WHO, 2017).

In conclusion, this is the first report to our knowledge to address resistance phenotypes of FIB from IRFs, which are an important conduit of surface waters in agroecosystems. The results indicate that the IRFs are polluted with material of fecal origin, which would not be surprising given that this is a mixed-use watershed and livestock manures are commonly applied to cropland soils. However, some of the *E. coli* and enterococcal isolates could be from naturalized extraintestinal populations that were released from sediments and soils. Regardless of FIB source, a wide variety of resistance patterns were found among many of the isolates, suggesting the potential for horizontal transfer of ARGs in the aquatic environment. Although *E. coli* and enterococci are intrinsically resistant to some of the antimicrobials tested, it may be possible that AMR among the isolates has also emerged in response to selection from the presence of antimicrobial residues or other chemical agents. While the FIB levels did increase during the irrigation season, resistant isolates were obtained during each sampling event throughout the year, indicating a permanent presence of ARB in the watershed. Since the IRFs do discharge into the Snake River, there is a potential opportunity for human contact with resistant *E. coli* and enterococci when the river is used for recreational purposes. Additional monitoring, as well as source tracking, of *E. coli* and enterococci in the IRFs will be necessary to understand the long-term trends of AMR and sources of FIB in this mixed-use watershed.

## Data Availability Statement

The raw data supporting the conclusions of this article will be made available by the authors, without undue reservation.

## Author Contributions

RD conceived and designed the study, analyzed the data, and wrote the manuscript. DB provided technical support and reviewed the manuscript. Both authors contributed to the article and approved the submitted version.

## Conflict of Interest

The authors declare that the research was conducted in the absence of any commercial or financial relationships that could be construed as a potential conflict of interest.

## Footnotes

1 https://www.fda.gov/media/108180/download
